# A Catalytic Role for Mod5 in the Formation of the Tea1 Cell Polarity Landmark

**DOI:** 10.1016/j.cub.2010.08.035

**Published:** 2010-10-12

**Authors:** Claudia C. Bicho, David A. Kelly, Hilary A. Snaith, Andrew B. Goryachev, Kenneth E. Sawin

**Affiliations:** 1 Wellcome Trust Centre for Cell Biology, University of Edinburgh, Edinburgh EH9 3JR, UK; 2 Centre for Systems Biology, University of Edinburgh, Edinburgh EH9 3JR, UK

## Abstract

Many systems regulating cell polarity involve stable landmarks defined by internal cues [1–5]. In the rod-shaped fission yeast *Schizosaccharomyces pombe*, microtubules regulate polarized vegetative growth via a landmark involving the protein Tea1 [6–9]. Tea1 is delivered to cell tips as packets of molecules associated with growing microtubule ends [10] and anchored at the plasma membrane via a mechanism involving interaction with the membrane protein Mod5 [11, 12]. Tea1 and Mod5 are highly concentrated in clusters at cell tips in a mutually dependent manner, but how the Tea1-Mod5 interaction contributes mechanistically to generating a stable landmark is not understood. Here, we use live-cell imaging, FRAP, and computational modeling to dissect dynamics of the Tea1-Mod5 interaction. Surprisingly, we find that Tea1 and Mod5 exhibit distinctly different turnover rates at cell tips. Our data and modeling suggest that rather than acting simply as a Tea1 receptor or as a molecular “glue” to retain Tea1, Mod5 functions catalytically to stimulate incorporation of Tea1 into a stable tip-associated cluster network. The model also suggests an emergent self-focusing property of the Tea1-Mod5 cluster network, which can increase the fidelity of polarized growth.

## Highlights

► Interacting polarity proteins Tea1 and Mod5 have different mobilities at cell tips ► Modeling suggests Tea1 assembles into polymeric cluster networks ► Mod5 acts catalytically in formation of Tea1 cluster networks ► Self-focusing property of cluster networks increases fidelity of polarized growth

## Results and Discussion

Tea1 is necessary for accurate restoration of the growth machinery to cell tip centers after stresses that perturb the actin cytoskeleton [6, 7, 11, 13]. This depends on Tea1 interactions with multiple downstream proteins involved in positioning polarized growth, including Bud6, Tea3, Tea4, and the formin For3 [12, 14–16]. Orientation of dynamic microtubules along the long axis of the cell normally biases delivery of Tea1 toward cell tips [8, 17]. However, occasional aberrant deposition of Tea1 could lead to undesirable spreading of the Tea1 polarity landmark unless additional factors ensure that only the Tea1 delivered close to cell tip centers becomes “anchored” at the plasma membrane. Because Mod5 is required for Tea1 anchoring at cell tips, and Tea1 is reciprocally required to restrict Mod5 localization to cell tips, it has been proposed that mutually dependent localization of Tea1 and Mod5 acts as a spatial positive-feedback loop in which Mod5 is specifically enriched at the very sites where it is required for correct Tea1 anchoring (Figure S1A, available online) [11, 12].

### Mod5 and Tea1 Have Different Turnover Rates within Cell Tips

**Figure 1:**
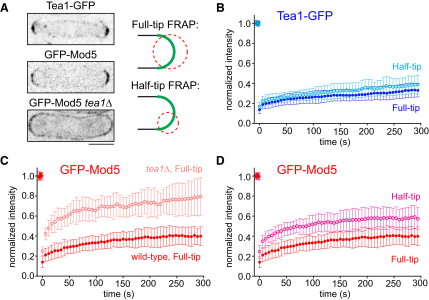
Mod5 Turns over More Quickly Than Tea1 within Cell Tips (A) Localization of Tea1-GFP and GFP-Mod5 in wild-type and *tea1Δ* cells, showing regions bleached in full-tip and half-tip FRAP experiments. Scale bar represents 5 μm. Fluorescence recovery of (B) Tea1-GFP after full-tip (n = 20) and half-tip (n = 13) bleaching in wild-type cells; (C) GFP-Mod5 after full-tip bleaching in wild-type (n = 16) and *tea1Δ* (n = 19) cells; (D) GFP-Mod5 after full-tip and half-tip (n = 19) bleaching in wild-type cells. GFP-Mod5 shows increased recovery after half-tip bleaching. For clarity the same full-tip trace is duplicated in (C) and (D). Error bars show standard deviations. See also Figure S1 and Table S1.

The simplest model in which the Tea1-Mod5 interaction could simultaneously account for both Tea1 anchoring and Mod5 enrichment at cell tips is one in which Tea1 and Mod5 interact stoichiometrically in a conventional ligand-receptor relationship, to generate relatively immobile Tea1-Mod5 complexes. This predicts that within such complexes, Tea1 and Mod5 should have identical mobility on the membrane. To test this, we used fluorescence recovery after photobleaching (FRAP) to measure the turnover of Tea1-GFP and GFP-Mod5 at cell tips (Figure 1). After bleaching entire tips (“full-tip FRAP”) of wild-type cells, we observed little recovery of either Tea1-GFP or GFP-Mod5 within the first 5 min (Figures 1A–1C). Thus, on this timescale both Tea1 and Mod5 are relatively stable at tips, with negligible exchange between tips and the cytoplasm or more medial membrane regions. In full-tip FRAP experiments of GFP-Mod5 in *tea1Δ* cells, we found a high degree of GFP-Mod5 turnover (Figure 1C). This suggests that when Tea1 is absent, Mod5 can freely diffuse in the membrane, consistent with the homogeneous distribution of GFP-Mod5 observed in *tea1Δ* cells (Figure 1A) [11, 12].

Interestingly, when we bleached only one-half of each cell tip (“half-tip FRAP”), GFP-Mod5 recovered much faster than Tea1-GFP (Figures 1B and 1D). Within 5 min, GFP-Mod5 recovered to approximately half of its initial value, as would be expected after mixing of bleached and unbleached GFP-Mod5 at the tip. By contrast, in half-tip FRAP, Tea1-GFP exhibited the same slow recovery as in full-tip FRAP. In response to microtubule depolymerization, Tea1-GFP and untagged Tea1 disappeared from cell tips at near-identical rates, indicating that slow FRAP recovery of Tea1-GFP is not an artifact of GFP-tagging (Figures S1C and S1D). These experiments demonstrate that even when membrane-associated Mod5 is restricted to cell tips, as in wild-type cells, Mod5 is considerably more dynamic than Tea1, invalidating the simple ligand-receptor model.

### Mod5 as a Catalyst for Tea1 Polymerization

**Figure 2:**
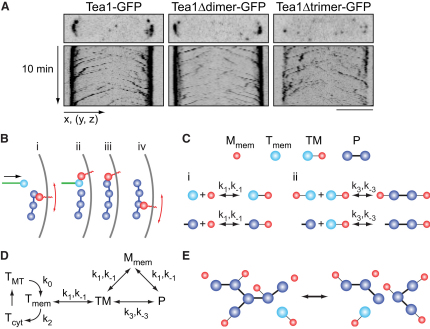
Interaction of Tea1 and Mod5 in the Formation of Tea1 Cluster Networks (A) Images and kymographs showing delivery of Tea1-GFP, Tea1Δdimer-GFP, and Tea1Δtrimer-GFP to cell tips. Tea1Δtrimer fails to accumulate at cell tips. (B) Steps in Tea1 polymerization and Mod5 dynamics. Incoming Tea1 on microtubules is shown in light blue, with a microtubule in green. Tea1 associated with cluster networks is shown in dark blue. Mod5 is shown in red. Mod5 diffuses in the membrane (i) and promotes the incorporation of newly arrived Tea1 (ii, iii) but remains restricted to the tip region by diffusion-capture mechanism (iv). (C) Proposed interactions between Tea1 and Mod5 (i), and between Tea1 and Tea1 (ii), with associated rate constants. Tea1 associated with membranes but not with cluster networks is shown in light blue. (D) Kinetic scheme used for computational modeling. (E) Conceptual diagram of temporal evolution of Tea1 cluster network as a result of the creation and dissolution of the Tea1-Tea1 and Tea1-Mod5 bonds described in (C). Further details are in text. See also Figure S2 and Movie S1.

The focused spatial distribution and stability of Tea1 in wild-type cells implies that microtubule-delivered Tea1 associates with a stable cortical structure. Based on the accumulation of Tea1 at cell tips in the form of adjacent and often overlapping nodes or clusters (Figure 2A; Movie S1), we will refer to this structure as a “cluster network.” In principle, Tea1 could either bind stably to a pre-existing polymeric network or form a network de novo. Because the cortical actin cytoskeleton, currently the only plausible candidate for a pre-existing network, is dispensable for Tea1 stability (Figures S2A and S2B) [6], we hypothesized that a polymeric Tea1 network may form de novo. Although network formation could involve additional Tea1-interacting proteins [12, 14–16], Tea1 is also known to interact with itself (Figure S2F) [16]. To extend over a two-dimensional area, a network must have local connectivity n ≥ 3 (see Experimental Procedures). This motivated us to mutate a region of Tea1 that is strongly predicted to form a trimeric coiled-coil (Figure S2C) [18]. Mutant Tea1 lacking the trimerization sequence was delivered by microtubules and interacted with Mod5 but failed to accumulate at cell tips in vivo, whereas mutation of an adjacent dimeric coiled-coil had no effect (Figure 2A; Figures S2D and S2E). Thus, the “trimer” region of Tea1 may contribute specifically to the formation of the Tea1 cluster network.

How can we reconcile the different dynamics of Tea1 and Mod5 with their localization interdependence? The dynamic behavior of Mod5 can be explained by Mod5 physically interacting only transiently with the Tea1 cluster network. Through frequent binding and unbinding, Mod5 can remain highly enriched but nevertheless mobile within the tip, via a diffusion-capture mechanism [19–21]. This, however, leaves unanswered the question of how Mod5 contributes mechanistically to the formation of stable Tea1 cluster networks. If the Tea1-Mod5 interaction is transient, it cannot play an integral structural role, e.g., as a molecular “glue” that links Tea1 molecules. We thus hypothesized that Mod5 does not merely tether Tea1 to the membrane but also acts to promote integration of individual incoming Tea1 molecules into the network (Figure 2B; Experimental Procedures).

We formalized these ideas in a minimal model for cluster-network assembly that explicitly postulates two types of reversible interactions with distinct properties: (1) readily formed but relatively unstable Tea1-Mod5 “bonds”; and (2) slowly formed but more stable Tea1-Tea1 “bonds” (Figure 2C; see Experimental Procedures for details). Because the model does not differentiate between direct or indirect Tea1-Tea1 bonds (e.g., via Tea1-interacting proteins), we do not make any further assumptions about their nature. In contrast to demonstrated Tea1-Tea1 and Tea1-Mod5 physical interactions, we found no evidence for Mod5-Mod5 interactions and therefore did not consider them further (Figure S2G) [12, 16]).

In the model, Tea1 and Mod5 associate to form polymeric networks with variable stoichiometry and connectivity (Figures 2C and 2E). Mod5 readily associates with and dissociates from these complexes by forming and breaking Tea1-Mod5 bonds. By contrast, incorporation of Tea1 into the network requires Tea1 to be presented by Mod5 in the context of the short-lived Tea1·Mod5 bimolecular complex. As with Tea1-Tea1 bonds, this hypothetical complex could include other auxiliary proteins. However, for the sake of simplicity, in the model Mod5 subsumes the functions of any such proteins (e.g., in the description below); this is justified provided that Mod5 (and Tea1) directly recruit these proteins into the complex. Because of the significant difference between the hypothesized lifetimes of the Tea1-Mod5 and Tea1-Tea1 bonds (see Table S2), a single molecule of Mod5 can promote sequential incorporation of multiple Tea1 molecules into the cluster network, through repeated rounds of Tea1·Mod5 incorporation and Mod5 dissociation (see Figure 4A). Because this occurs without “consumption” of Mod5, by definition Mod5 plays the role of a catalyst for Tea1 polymerization. As required by thermodynamic reversibility, removal of individual Tea1 molecules from the polymer occurs through the breakage of Tea1-Tea1 bonds, via dissociation of Tea1·Mod5 complexes. Mod5, like any other catalyst, also accelerates this reaction, i.e., depolymerization of the Tea1 network (see Figure 4B). In the context of a kinetic scheme including microtubule-based delivery of Tea1 and its recycling back to the cytoplasm (Figure 2D), repeated formation and dissociation of Tea1-Mod5 and Tea1-Tea1 bonds can account for all types of cluster-network dynamics at cell tips, including local growth, dissolution, and changes in connectivity accompanied by rearrangements of clusters within the overall cluster network (Figure 2E).

Microscopic features of the model, such as the specific connectivity of Tea1 in a polymeric network or the existence of Tea1·Mod5 intermediates, cannot be directly validated with currently available experimental tools. However, the model can be used to make predictions that are testable at the whole-cell level. To convert the proposed mechanism into a predictive computational model, we constrained its parameters by using experimental data (Experimental Procedures). By immunoblotting, we measured approximately 8000 Tea1 and 2000 Mod5 molecules per cell (Figures S3A and S3B), and by fluorescence imaging we measured 1510 ± 500 (SD) Tea1 and 380 ± 200 (SD) Mod5 molecules per cell tip (Figure S3C). Using time-lapse videomicroscopy, we found that microtubule-deposited Tea1 packets contain, on average, 80 Tea1 molecules. Because the model suggested significant turnover of Tea1 on longer timescales, we repeated Tea1-GFP FRAP and GFP-Mod5 FRAP on these timescales (Figures S3E and S3F) and used the results to further constrain model parameters (Table S2).

**Figure 3:**
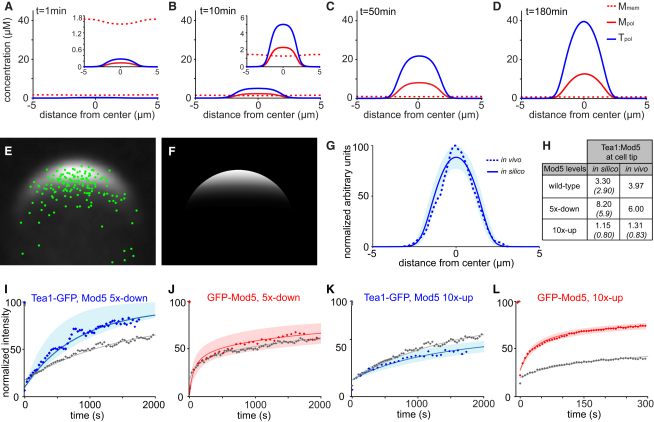
In Silico Simulations Recapitulate Tea1 Landmark Formation and Accurately Predict System Dynamics (A–D) Time evolution of Tea1 cluster-network formation at cell tips, showing local concentration of Tea1 incorporated into cluster networks (Tpol), Mod5 bound to cluster networks (Mpol), and free Mod5 (Mmem). The x axis indicates distance along cell perimeter. (E) Population-averaged distribution of Tea1-GFP at cell tips in wild-type cells, calculated from 60 individual images. Green dots indicate positions of Tea1-GFP deposition by microtubules from video sequences (211 events, from eight cell tips). Note Tea1 deposition events far from the cell tip center. (F) In silico steady-state Tea1 distribution. (G) Normalized steady state Tea1-GFP fluorescence at the cell tip, measured from (E) (dashed line) and (F) (solid line). Shaded area represents 95% confidence interval for model prediction (see Experimental Procedures). (H) Predicted in silico and experimental Tea1:Mod5 cell tip ratios. Values without parentheses refer to total Tea1 and Mod5 at tips, and values in parentheses refer to cluster-network-associated Tea1 and Mod5 at tips. (I–L) Fluorescence recovery after full-tip photobleaching of (I) Tea1-GFP (n = 8), (J) GFP-Mod5 (n = 13), (K) Tea1-GFP (n = 11), and (L) GFP-Mod5 (n = 11) in cells with altered Mod5 expression. In (I)–(L), solid lines represent in silico predictions and dots represent in vivo measurements. Shaded areas indicate confidence intervals of model predictions estimated from parameter variation (Experimental Procedures). For comparison, data (dots) and model simulations (lines) for wild-type Mod5 expression (Figure S3) are shown in gray. See also Figure S3 and Table S2.

We then tested the ability of the model to recreate steady-state distributions of Tea1 and Mod5 at cell tips in silico. Starting from an initial state of Tea1 in the cytoplasm and Mod5 uniformly distributed on the membrane, simulations delivering Tea1 to cell tips at physiological rates recapitulated the de novo formation of cell-tip cluster networks observed experimentally upon microtubule regrowth after prolonged depolymerization (Figures 3A–3D) [6]. Similar results were obtained whether Tea1 was delivered to the cell tips stochastically as packets, as occurs in vivo, or as a continuous flux (Experimental Procedures). Overall, these simulations led to steady-state levels of approximately 1200 Tea1 and 400 Mod5 molecules per cell tip, in good agreement with experimental measurements. Moreover, the concentration profile of Tea1 across the cell tip averaged over multiple cells was similar to the profile predicted by the model (Figures 3E–3G).

In contrast to the simple receptor-ligand mechanism with a fixed Tea1:Mod5 ratio, our model predicts that changes in their expression levels will alter their stoichiometry at cell tips. To test this, we generated strains with increased and decreased Mod5 expression (data not shown) and calculated predicted Tea1:Mod5 tip ratios and full-tip FRAP curves for both GFP-Mod5 and Tea1-GFP. Results of experiments using these strains showed good agreement with model predictions, for both increased and decreased Mod5 expression, and computed confidence intervals demonstrate that the model is robust to parameter variation (Figures 3H–3L). Both experiment and theory demonstrate the exquisite robustness and flexibility of the Tea1-Mod5 system in providing a functional Tea1 landmark over a 50-fold range of Mod5 expression.

### Increased Robustness via Self-Focusing

**Figure 4:**
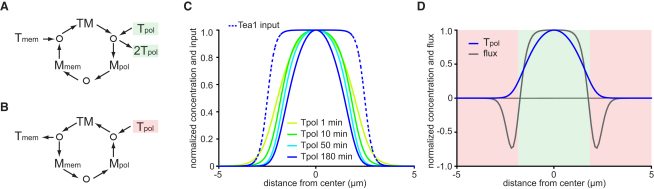
Tea1 Cluster Networks Exhibit Self-Focusing Behavior (A and B) Directionality of Tea1 cluster-network polymerization depends on the local concentration of polymeric Tea1. In regions with high polymeric Tea1, autocatalysis drives net Tea1 polymerization, and the Mod5 “cycle” proceeds in a clockwise direction (A; green zone in D). In regions with low polymeric Tea1 there is net Tea1 depolymerization, and the Mod5 cycle proceeds anticlockwise (B; red zone in D). (C) Normalized concentration of polymeric Tea1 during de novo network formation as predicted by the model, shown together with the normalized profile of Tea1 deposition by microtubules. The cluster network becomes progressively more focused over time. The x axis indicates distance along cell perimeter. (D) Normalized Tpol concentration and polymerization reaction flux at steady state. The x axis is as in (C). See also Figure S4.

In both experiments and simulations, the rate of recovery of Tea1 after FRAP was slower than would be expected from steady-state rate of Tea1 delivery by microtubules, indicating that a significant amount of delivered Tea1 is not actually incorporated at cell tips. We found that on average, only ∼33% of microtubule-delivered Tea1 is incorporated into the cluster network, with the remainder recycled back to the cytoplasm. Analysis of the model revealed that Tea1 delivered to the center of a cluster network incorporates into the network with more than twice average probability, whereas Tea1 delivered to the periphery incorporates with lower than average probability (Figure S4A). These differences in incorporation result from the fact that in the proposed kinetic scheme, Mod5-mediated Tea1 polymerization is an autocatalytic reaction (Figures 4A and 4B). Similar to Arp2/3-mediated actin branching [22] the autocatalytic nature of Tea1 polymerization in the model arises from hypothesized network connectivity n ≥ 3. Incorporation of new Tea1 molecules creates additional sites of Tea1 polymerization, and thus the main driver of Tea1 accumulation is the magnitude of the local concentration of polymeric Tea1 itself, and not the local concentration of Mod5 as was proposed by us initially [11]. Indeed, although total Mod5 is enriched at cell tips, most of it is bound to the cluster network, and thus at any given time, the concentration of free Mod5 (i.e., Mod5 available to promote incorporation of Tea1) remains almost uniform across the membrane (Figures 3A–3D).

Could apparently inefficient incorporation nevertheless have benefits for Tea1 landmark function? We found that autocatalytic polymerization of membrane-bound Tea1, coupled with its recycling back to the cytoplasm, results in the emergent property of cluster-network self-focusing. Simulations of de novo cluster-network formation showed that despite an increase in the overall amount of network-associated Tea1 (Figures 3A–3D), the width of the cluster network continuously decreases until it reaches steady state (Figure 4C). Some decrease in network width is observed even after the total amount of membrane-bound Tea1 reaches a plateau (Figures S4C and S4D). Profiles of polymeric Tea1 concentration and reaction flux reveal that even at steady state, the cluster network is highly dynamic; it is continuously assembled in the center and disassembled at the periphery (Figure 4D; see Experimental Procedures). This self-focusing is opposed by diffusion, and, at steady state, the contributions of these two effects are fully balanced (see also Figure S4B).

Self-focusing implies that the region of polymerized Tea1 will generally be narrower than the Tea1 “delivery zone” defined by microtubule dynamics (Figures 3E and 4C). From the viewpoint of Tea1 landmark function, this offers two potential benefits. First, during de novo formation, self-focusing provides a means for the cluster network to “find” the center of the microtubule delivery zone and thus narrow the zone of polarized growth. Consistent with this, even when they do not develop gross polarity defects, *tea1*Δ cells are generally wider than their wild-type counterparts [23]. Second, at steady state, self-focusing offers robustness to microtubule errors, because Tea1 packets delivered aberrantly far from the cluster-network center will rapidly recycle back to the cytoplasm, whereas those deposited close to the center will reinforce the network. The cluster network thus possesses significant “inertia,” allowing it to maintain its position, and therefore that of the cell-growth machinery, even in the presence of inevitable noise due to fluctuations in microtubule dynamics. In effect, the cluster network filters out all perturbations to the microtubule cytoskeleton with characteristic times shorter than its own lifetime, in agreement with our finding that after microtubule depolymerization, Tea1-GFP dissipates slowly from cell tips, over tens of minutes (Figures S4E and S4F). More broadly, we speculate that the ability of the microtubule-Tea1-Mod5 system to find and maintain the tip center may be just as important as microtubule-based Tea1 delivery. From a design viewpoint, the increased overall fidelity and robustness to perturbation of the system is “paid for” by relatively inefficient average incorporation of Tea1.

## Conclusions

Recent studies modeling fission yeast cytoskeleton and cell polarity have provided useful insights into the mechanisms underlying complex behaviors [24, 25]. Our model demonstrates how Tea1 and Mod5 can dynamically generate a polarity landmark that is robust to perturbation. The principles described here may apply more generally to microtubule-delivered landmark systems controlling localized growth and cytoskeleton remodeling in other eukaryotes, even where they bear no superficial resemblance to the Tea1-Mod5 system. Possible examples include adenomatous polyposis coli (APC) clusters organized by microtubules in migrating mammalian cells [26, 27], and microtubule-dependent activation of RhoA GTPase at the equatorial membrane prior to cytokinesis in metazoan cells [28, 29].

Mechanistically, Mod5 appears to play a role similar to that of small GTPases involved in assembly of polymeric protein complexes such as vesicle transport coats [30]. During coat assembly, recycling of Arf GTPases allows them to perform multiple rounds of coatomer recruitment and incorporation into polymer. Several features of the mechanism proposed here are also shared with those involved in forming a cluster of activated Rho GTPase Cdc42 at the yeast presumptive bud site [31, 32]. However, in contrast to small GTPases that actively consume energy to create structures, in the Tea1-Mod5 system both the symmetry-breaking stimulus and the required energy are provided externally by continuous microtubule polymerization.

## Supplemental Information

Document S1. Supplemental Experimental Procedures, Four Figures, and Two Tables

Movie S1. High Time-Resolution Imaging of Tea1-GFP Spinning-disk confocal imaging of Tea1-GFP in packets and at cell tips in a wild-type cell over a 5 min period (time compression ∼50×). Maximum projection of eight Z sections is shown. To increase signal, we intentionally kept illumination intensity high relative to conventional imaging; as a result some photobleaching is observed. Note that Tea1 packets appear to lose individual identity over time. We interpret variations in intensity at cell tips as the superposition of incoming Tea1 packets and dynamic rearrangements of Tea1 in cluster networks (see main text).
