# Supplementary material for: A Catalytic Role for Mod5 in the Formation of the Tea1 Cell Polarity Landmark
Source: Curr Biol. 2010 Oct 12;20(19):1752–7. doi: 10.1016/j.cub.2010.08.035 (PMC3094757; doi:10.1016/j.cub.2010.08.035)
Supplement: Document S1. Supplemental Experimental Procedures, Four Figures, and Two Tables [file mmc1.pdf]

Supplemental Information
Current Biology, Volume *20*

A Catalytic Role for Mod5 in the Formation of the Tea1 Cell Polarity Landmark

Claudia C. Bicho, David A. Kelly, Hilary A. Snaith, Andrew B. Goryachev, and Kenneth E. Sawin

Figure S1

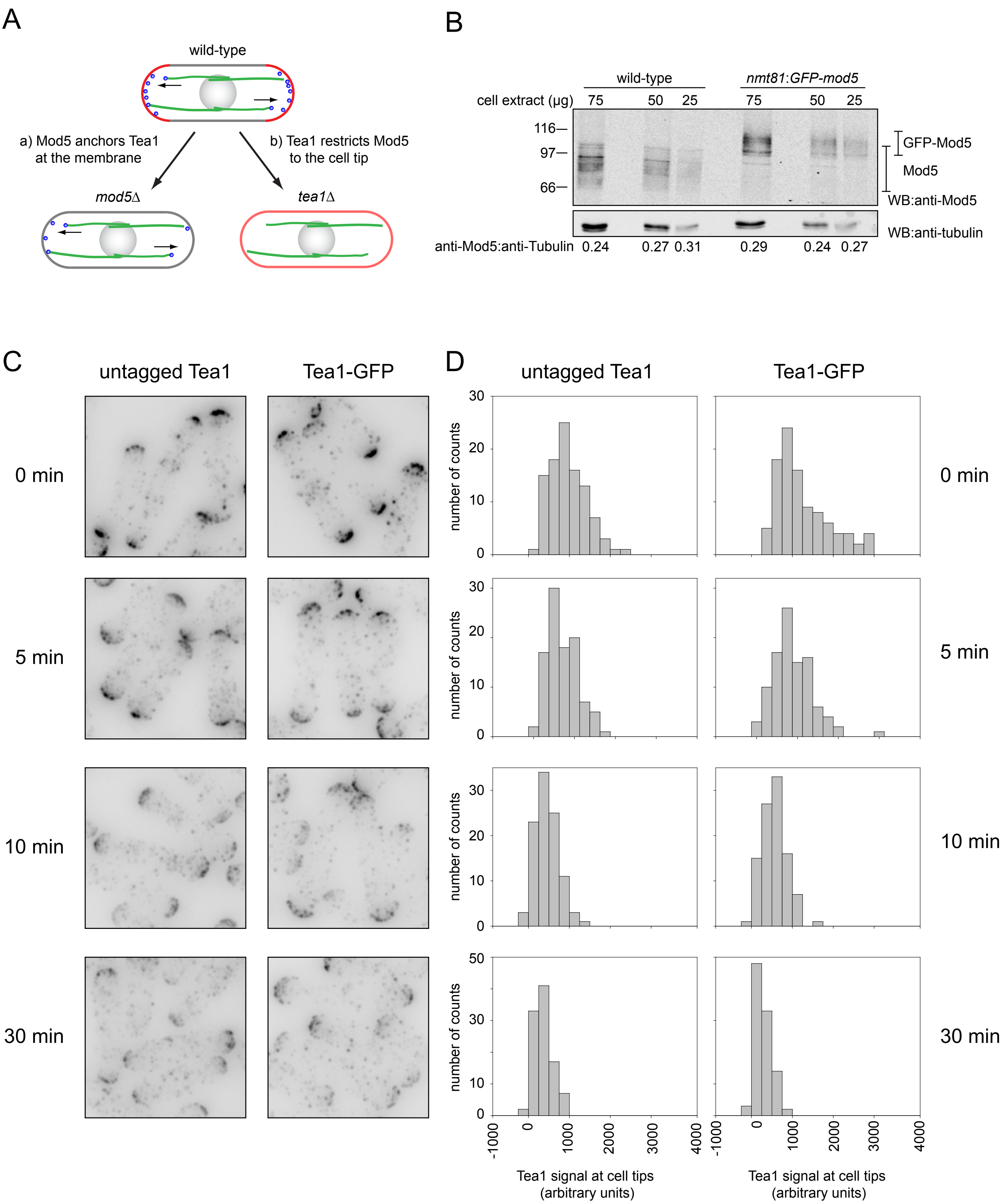
A
wild-type
a) Mod5 anchors Tea1 at the membrane
b) Tea1 restricts Mod5 to the cell tip
mod5Δ
tea1Δ
B
wild-type
nmt81:GFP-mod5
cell extract (μg) 75 50 25 75 50 25
116—
97—
66—
GFP-Mod5
Mod5
WB:anti-Mod5
WB:anti-tubulin
anti-Mod5:anti-Tubulin 0.24 0.27 0.31 0.29 0.24 0.27
C
untagged Tea1
Tea1-GFP
0 min
5 min
10 min
30 min
D
untagged Tea1
Tea1-GFP
number of counts
Tea1 signal at cell tips (arbitrary units)

Figure S2

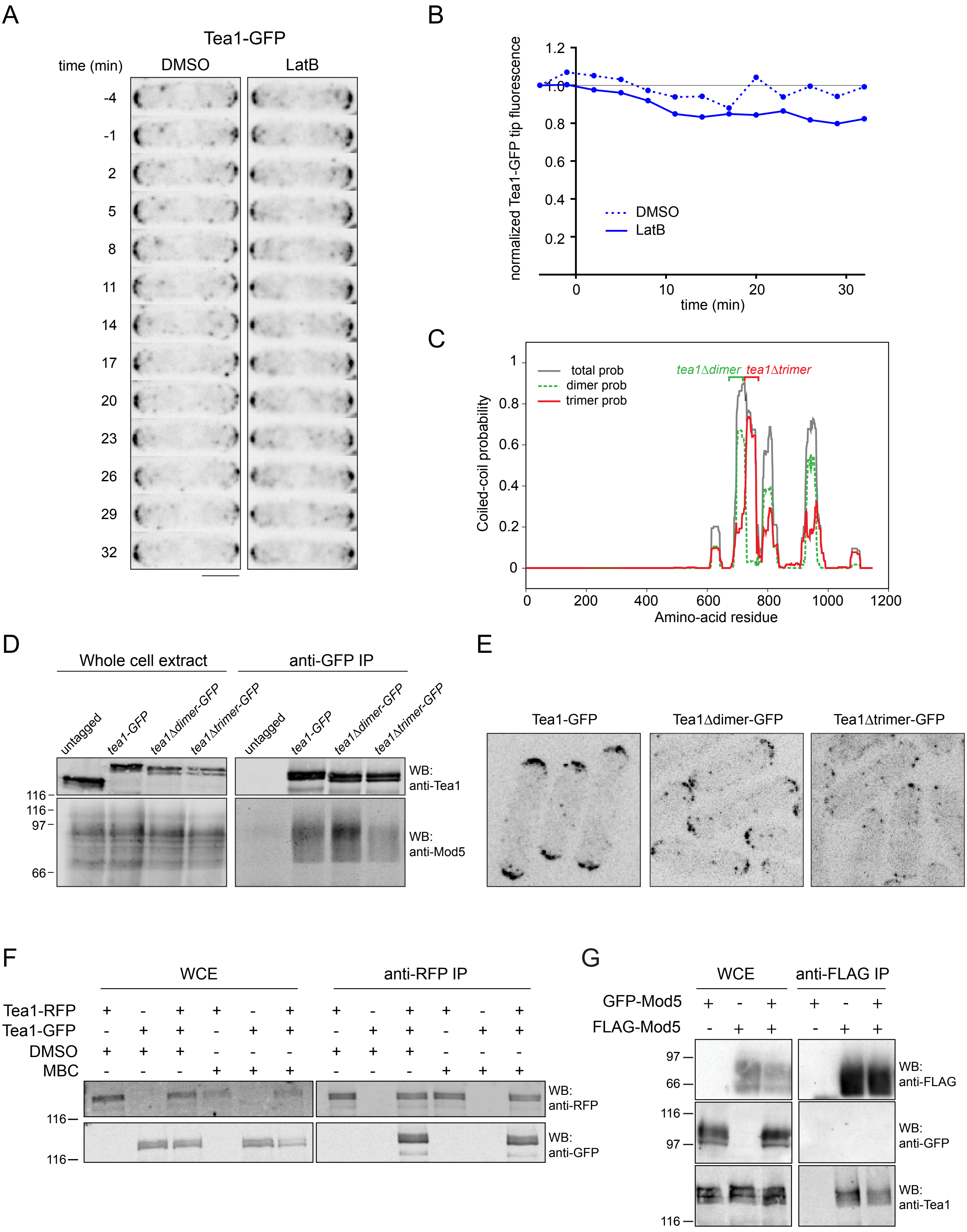
A
Tea1-GFP
time (min)
DMSO
LatB
-4
-1
2
5
8
11
14
17
20
23
26
29
32
B
normalized Tea1-GFP tip fluorescence
time (min)
DMSO
LatB
C
Coiled-coil probability
Amino-acid residue
total prob
dimer prob
trimer prob
tea1Δdimer
tea1Δtrimer
D
Whole cell extract
anti-GFP IP
untagged
tea1-GFP
tea1Δdimer-GFP
tea1Δtrimer-GFP
WB: anti-Tea1
WB: anti-Mod5
E
Tea1-GFP
Tea1Δdimer-GFP
Tea1Δtrimer-GFP
F
WCE
anti-RFP IP
Tea1-RFP
Tea1-GFP
DMSO
MBC
WB: anti-RFP
WB: anti-GFP
G
WCE
anti-FLAG IP
GFP-Mod5
FLAG-Mod5
WB: anti-FLAG
WB: anti-GFP
WB: anti-Tea1

Figure S3

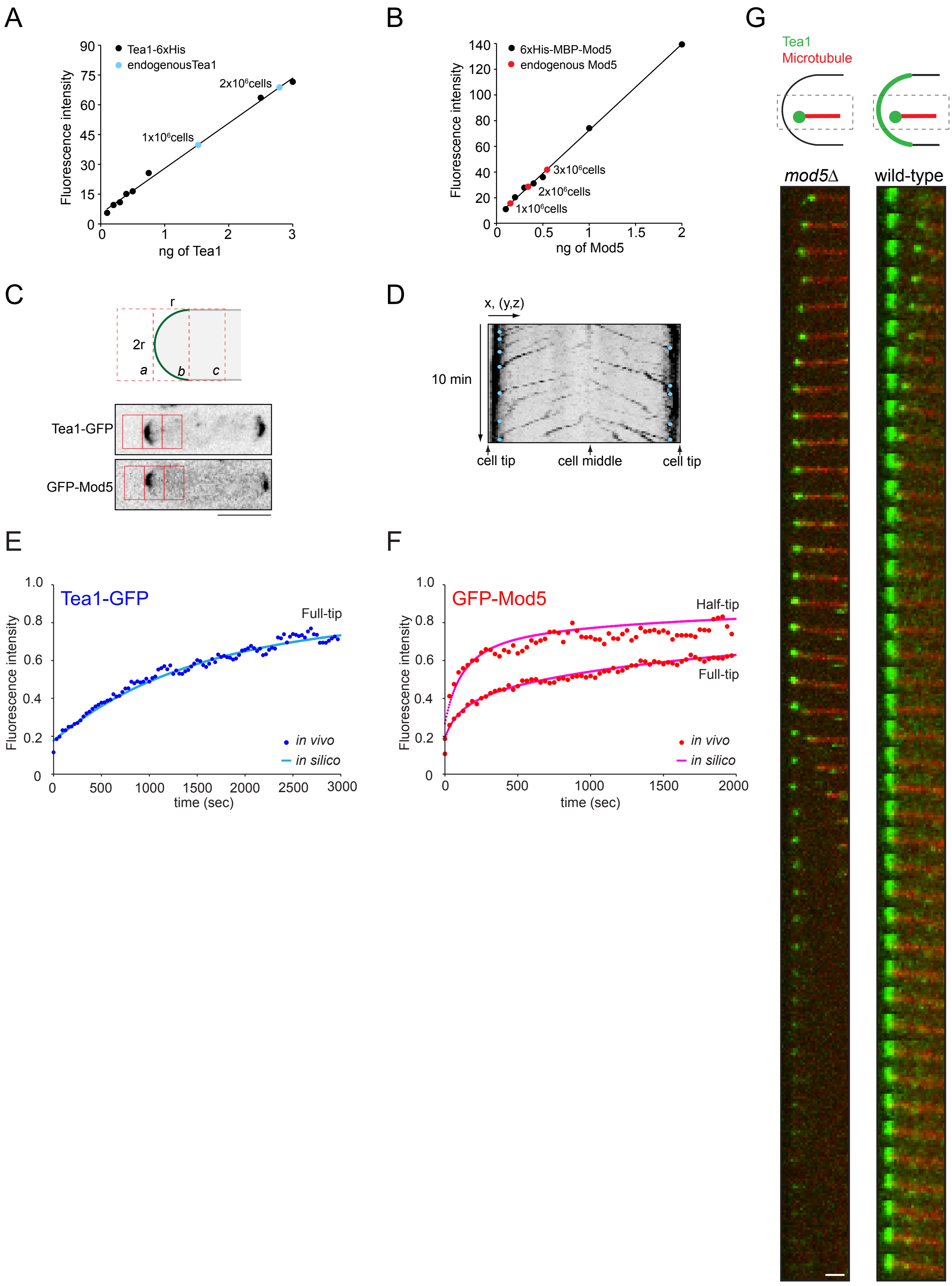
A
Fluorescence intensity
ng of Tea1
Tea1-6xHis
endogenousTea1
2x10⁶cells
1x10⁶cells
B
Fluorescence intensity
ng of Mod5
6xHis-MBP-Mod5
endogenous Mod5
3x10⁶cells
2x10⁶cells
1x10⁶cells
C
r
2r
a
b
c
Tea1-GFP
GFP-Mod5
D
x, (y,z)
10 min
cell tip
cell middle
cell tip
E
Tea1-GFP
Full-tip
Fluorescence intensity
time (sec)
in vivo
in silico
F
GFP-Mod5
Half-tip
Full-tip
Fluorescence intensity
time (sec)
in vivo
in silico
G
Tea1
Microtubule
mod5Δ
wild-type

Figure S4

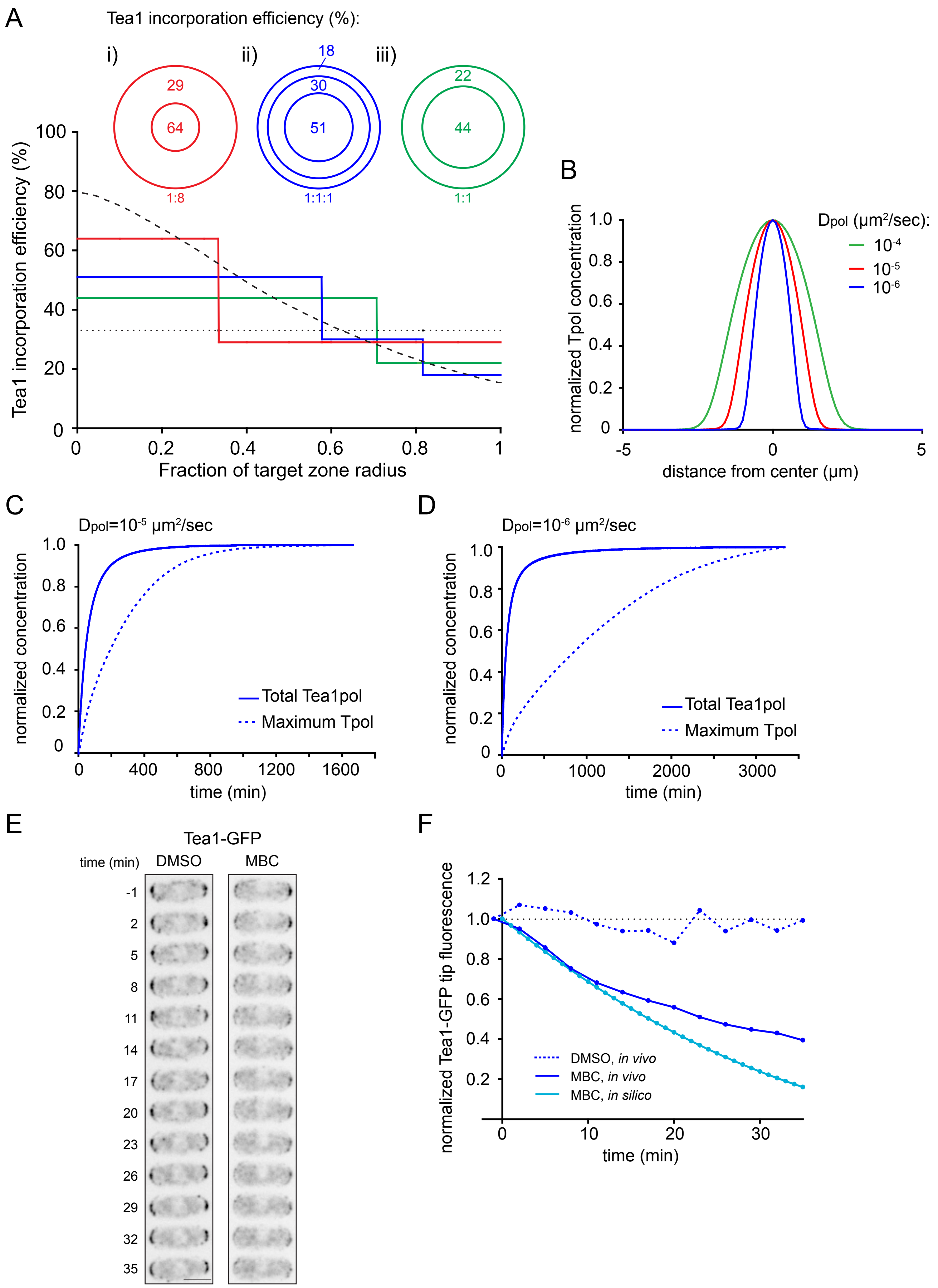
A
Tea1 incorporation efficiency (%):
i)
ii)
iii)
29
64
1:8
18
30
51
1:1:1
22
44
1:1
Tea1 incorporation efficiency (%)
Fraction of target zone radius
B
normalized Tpol concentration
Dpol (μm²/sec):
10-4
10-5
10-6
distance from center (μm)
C
Dpol=10-5 μm²/sec
normalized concentration
Total Tea1pol
Maximum Tpol
time (min)
D
Dpol=10-6 μm²/sec
normalized concentration
Total Tea1pol
Maximum Tpol
time (min)
E
Tea1-GFP
time (min)
DMSO
MBC
-1
2
5
8
11
14
17
20
23
26
29
32
35
F
normalized Tea1-GFP tip fluorescence
DMSO, in vivo
MBC, in vivo
MBC, in silico
time (min)

SUPPLEMENTAL FIGURE LEGENDS

Figure S1, related to Figure 1.

Tea1 and Mod5 localization and behavior.

(A) Cartoon of Tea1 and Mod5 localization in wild-type cells and in *mod5*Δ and *tea1*Δ cells. (B) Expression levels of *nmt81*:GFP-Mod5 grown in 150 nM thiamine are identical to wild-type. Wild-type and *nmt81:GFP-mod5* cells were grown in 150 nM thiamine, and cell extracts were loaded in a dilution series for quantitative western blotting. Anti-Mod5 signals relative to anti-tubulin (loading control) are shown underneath. (C) anti-Tea1 immunofluorescence images showing effects of MBC-induced microtubule depolymerization on Tea1 localization in wild-type cells and *tea1-GFP* cells over time. No difference was observed between the two strains, indicating that GFP-tagging of Tea1 does not significantly alter Tea1 turnover at cell tips. (D) Quantitation of Tea1 and Tea1-GFP immunofluorescence at cell tips for the experiment shown in (C). For each histogram 100 tips were scored (see Experimental Procedures).

Figure S2, related to Figure 2.

Evidence for *de novo* formation of Tea1 cluster-networks.

(A) Time-course of Tea1-GFP distribution in a cell treated with 200 μM latrunculin B (LatB) to disrupt the actin cytoskeleton, or with DMSO (solvent) alone. (B) Average normalized values of cell tip-associated Tea1-GFP in LatB-treated cells over time (19 cell tips), compared with DMSO-treated controls (25 cell tips). In (A) and (B), "zero" time is 7 minutes after drug addition, by which time all polymeric actin has disappeared (our unpublished data). (C) MultiCoil prediction [18] for Tea1. Amino acids 697-726 are predicted to form a dimeric coiled-coil, but amino acids 727-762 are predicted to form a trimeric coiled-coil. These two regions were independently deleted from Tea1 to generate Tea1Δdimer and Tea1Δtrimer. (D) Anti-Tea1 and anti-Mod5 western blots of anti-GFP immunoprecipitates from *tea1-GFP*, *tea1Δdimer-GFP* and *tea1Δtrimer-GFP* cells. Like Tea1 and Tea1Δdimer, Tea1Δtrimer binds to Mod5. (E) Live-cell imaging showing that unlike Tea1 and Tea1Δdimer, Tea1Δtrimer does not accumulate at cell tips. (F) Tea1 interacts with itself. Anti-GFP and anti-RFP western blots showing co-immunoprecipitation of Tea1-GFP with co-expressed Tea1-RFP. Note that interaction occurs equally after disruption of microtubules with methyl-benzimidazoyl-carbamate (MBC, 262 μM) as with DMSO (solvent) alone, when Tea1 is not at cell tips, and thus these data *per se* are not unequivocal "proof" for Tea1 polymerization. (G) Mod5 does not interact with itself. Western blots showing absence of co-immunoprecipitation of GFP-Mod5 with FLAG-Mod5. In the same experiment, Tea1 is co-immunoprecipitated with FLAG-tagged Mod5.

Figure S3, related to Figure 3.

Experimental data used to parametrize the cluster-network model .

(A) Amounts of Tea1 in fission yeast cell extracts, based on quantitative western blotting and calibration with recombinant Tea1-6xHis. (B) Amounts of Mod5 in fission yeast cell extracts, based on quantitative western blotting and calibration with recombinant 6xHis-MBP-Mod5. (C) Strategy for measurements of Tea1-GFP and GFP-Mod5 fluorescence at cell tips (see Experimental Procedures for details). Bar, 5 μm. (D) Kymograph showing microtubule-based delivery of Tea1-GFP to cell tips over a 10-minute time period. Deposition of representative packets is shown in blue. (E) Experimental data (dots) and computer simulation (line) of long-term fluorescence recovery of Tea1-GFP after full-tip bleaching in wild-type cells. (F) Experimental data (dots) and computer simulation (line) of long-term fluorescence recovery of GFP-Mod5 after full-tip and half-tip bleaching in wild-type cells. Fit between experiment and theory in (E) and (F) were used to further constrain model parameters. (G) Residence of Tea1-RFP (shown here in green) at the cell-tip membrane in *mod5*Δ cells, after deposition of a Tea1 packet by GFP-labelled microtubules (shown in red). Wild-type images were acquired identically and are shown as a photobleaching control. Cartoon indicates the portions of the cell tips shown in the figure. Images were taken at 5.5 sec intervals. Bar, 1 μm. The average packet residence time in such experiments was 115 sec (n = 7).

Figure S4, related to Figure 4.

Self-focusing and frequency-filtering properties of the Tea1 cluster-network.

(A) Tea1 incorporation efficiency in different concentric sub-zones of the microtubule-targeting zone, based on *in silico* simulation (see Experimental Procedures for details). i) Targeting zone divided into central disc and surrounding annulus, with area ratio 1:8. ii) Targeting zone divided into central disc and two surrounding annuli, with area ratio 1:1:1. iii) Targeting zone divided into central disc and surrounding annulus, with area ratio 1:1. Numbers inside sub-zones indicate average Tea1 incorporation efficiency within that sub-zone. Stepped lines in graph show average values of Tea1 incorporation efficiency for the different sub-zones as a function of the fractional radius of the sub-zone relative to the target zone, color-coded as in i, ii and iii. Dotted line represents average Tea1 incorporation efficiency over the entire target zone. Dashed line represents approximately estimated Tea1 incorporation efficiency as a continuous function of distance from center of target zone. (B) Self-focusing of the Tea1 cluster-network increases as the diffusion coefficient of polymeric Tea1 decreases. Plots show normalized local concentration of Tea1 incorporated into cluster-networks (i.e. T_{pol} in Figs. 3,4) at steady state, in simulations with different diffusion coefficients D_{pol}. X-axis indicates distance from the cell tip center along cell perimeter. (C, D) Continued increase in maximum concentration of polymeric Tea1 (i.e. local concentration of T_{pol} at center of microtubule-targeting zone; dashed line) even after total polymeric Tea1 (solid line)

reaches a plateau. Conservation of mass implies that the width of the cluster-network must simultaneously decrease, indicating that at later stages, polymerization in the cluster-network center occurs solely at the expense of depolymerisation at the network periphery. This effect is particularly striking when diffusion coefficient D_{pol} is low (D). (E) Time-course showing decrease in cell tip-associated Tea1-GFP after treatment with 262 μM methyl-benzimidazoyl-carbamate (MBC) to disrupt microtubule-based targeting to cell tips, compared to DMSO (solvent) alone. (F) Average normalized values of cell tip-associated Tea1-GFP in MBC-treated cells over time (33 cell tips), relative to DMSO-treated controls (25 cell tips; data duplicated from Fig. S2B). "MBC *in silico*" indicates calculated decay of total cluster network-associated Tea1 after cessation of microtubule-based Tea1 delivery. Note good agreement of experimental and theoretical curves at early stage of decay. Later deviation may be attributable to weak interactions of Tea1 with various membrane-associated molecules. "Zero" time in (E) and (F) is 4 minutes after MBC addition, by which time only a few microtubule remnants are found in cells, with none near cell tips (our unpublished data;[33]).

Movie S1, available separately online.
High time-resolution imaging of Tea1-GFP.
Spinning-disk confocal imaging of Tea1-GFP in packets and at cell tips in a wild-type cell over a five-minute period (time compression ~50X). Maximum projection of 8 Z-sections is shown. To increase signal, illumination intensity was intentionally kept high relative to conventional imaging; as a result there some photobleaching is observed. Note that Tea1 packets appear to lose individual identity over time. We interpret variations in intensity at cell tips as the superposition of incoming Tea1 packets and dynamic rearrangements of Tea1 in cluster-networks (see main text).

Table S1
Strains and plasmids used in this work

Plasmid	***Description***	***Reference***
pKS537	p0GWA	[34]
pKS544	pHMGWA	[34]
pKS818	Tea1 in pDUAL-GFH1c	This study
pKS914	Mod5 in pDUAL-HFF41c	This study
pKS1102	Tea1 in p0GWA	This study
pKS1103	Mod5(aa28-477) in pHMGWA	This study
pKS1104	pDUAL-GFH1c	[35]
pKS1105	pDUAL-HFF41c	[35]
pKS1025	*tea1Δ(aa697-726)* in pDONR221	This study
pKS1029	*tea1Δ(aa727-762)* in pDONR221	This study

Strain	***Genotype***	***Reference***
KS136	*h- tea1Δ::ura4+ ade6-M210 ura4-D18*	[6]
KS515	*h+ ade6-M216 leu1-32 ura4-D18*	Lab stock
KS516	*h- ade6-M210 leu1-32 ura4-D18*	Lab stock
KS706	*h- kanMX6:nmt81GFP-mod5 ade6-M210 leu1-32 ura4-D18*	[11]
KS714	*h- kanMX:nmt81:GFP- mod5 tea1Δ::ura4+ ade6-M216 leu1-32 ura4-D18*	[11]
KS780	*h+ mod5Δ::kanMX6 ade6-M216 leu1-32 ura4-D18*	[11]
KS1259	*h- tea1-GFP:kanMX ade6-M210 leu1-32 ura4-D18*	[11]
KS1260	*h+ tea1-GFP:kanMX ade6-M210 leu1-32 ura4-D18*	[11]
KS3138	*h- tea1-tdTomato:natMX6 ade6-210 leu1-32 ura4-D18*	This study
KS4417	*h+ tea1Δ::ura4+ leu1-32:p[leu1+:nmt1:tea1-GFP-FLAG-6His] ade6-M210 ura4-D18*	This study
KS4628	*h+ tea1-tdTomato:natMX6 hphMX6:nmt81GFP-atb2 ade6-M210 leu1-32 ura4-D18*	This study
KS4684	*h+ mod5Δ::kanMX6 tea1-tdTomato:natMX6 hphMX6:nmt81GFP-atb2 ade6-M210 leu1-32 ura4-D18*	This study
KS4774	*h? tea1-tdTomato:natMX6 leu1-32:p[leu1+:nmt1:tea1-GFP-FLAG-6His] ade6-M210 ura4-D18*	This study
KS4821	*h- mod5Δ::hphMX6 leu1-32:p[leu1+:nmt41:6His-FLAG2-mod5] ade6-M210 ura4-D18*	This study
KS4889	*h- kanMX6:nmt81GFP-mod5 leu1-32:p[leu1+:nmt41:6His-FLAG2-mod5] ade6-M210 ura4-D18*	This study
KS5661	*h+ tea1Δ(697-726aa)GFP:kanMX ade6-M210 leu1-32 ura4-D18*	This study
KS5980	*h+ tea1Δ(726-762aa)GFP:kanMX ade6-M210 leu1-32 ura4-D18*	This study
KS5620	*h+ natMX6:nmt81:mod5 tea1-GFP:KanMX ade6-M210 leu1-32 ura4-D18*	This study

Table S2
Parameters of the Tea1-Mod5 cluster-network model

**Constant**	**Value/range**	**Optimized Value***
k_0	$(0.009-0.02)\mu M\cdot s^{-1}$	$0.016\mu M\cdot s^{-1}$ or 116 molecules/packet
k_1	$(0.1-10)\,\mu M^{-1}\cdot s^{-1}$	$2\,\mu M^{-1}\cdot s^{-1}$
k_{-1}	$(0.1-10)\,s^{-1}$	$3\,s^{-1}$
k_2	$(0.007-0.7)\,s^{-1}$	$0.07\,s^{-1}$
k_3	$(0.01-0.9)\,\mu M^{-1}\cdot s^{-1}$	$0.21\,\mu M^{-1}\cdot s^{-1}$
k_{-3}	$(0.001-0.1)\,s^{-1}$	$0.03\,s^{-1}$
Tea1 *total*	8000 molecules/cell	
Mod5 *total*	2000 molecules/cell	
M^0_{mem}	$(0.72-2.4)\mu M$	$1.7\mu M$
D_m	$0.05\mu m^2\cdot s^{-1}$	
D_p	$(10^{-3}-10^{-6})\mu m^2\cdot s^{-1}$	$10^{-4}\,\mu m^2\cdot s^{-1}$

* Model parameters were varied in the indicated ranges to obtain optimal fit with the experimental data.

SUPPLEMENTAL EXPERIMENTAL PROCEDURES

General yeast methods

S. pombe methods were as described [36]. Expression of *nmt81*:GFP-Mod5 was induced for 2 x overnight at 25˚C in EMM2 plus 150 nM thiamine. Cultures were diluted 1:100 in EMM2 plus 150 nM thiamine and grown 1 x overnight at 25˚C prior to imaging. Quantitative western blotting showed that this produced levels of GFP-Mod5 very close to wild-type levels of untagged Mod5 (Fig. S1B). To vary Mod5 expression levels, strains expressing *nmt81*:Mod5 and *nmt81:*GFP-Mod5 were grown in the presence of 15 μM thiamine (Mod5 5x-down) or in the absence of thiamine (Mod5 10x-up) as described above. Tea1-GFP was co-expressed with Tea1-RFP, and FLAG-Mod5 with GFP-Mod5, by integration of *nmt1*:Tea1-GFP-FLAG-6xHis or *nmt41*:2xFLAG-6xHis-Mod5 at the *leu1* locus, using methods described in [35]. Tea1 co-expressing strains were grown in 15 μM thiamine to restore Tea1-GFP protein levels to similar to wild-type (data not shown). To construct *tea1Δdimer* and *tea1Δtrimer* strains, amino acids 697-726 and 727-762 of Tea1 were replaced, respectively, by a linker sequence containing five repeats of the dipeptide GlySer (GGTAGCGGAAGTGGTAGTGGAAGCGGCAG), at the genomic *tea1* locus. Molecular biology methods were essentially as described [37]. A list of plasmids and strains used in this work appear in Table S1.

Fluorescence imaging and fluorescence recovery after photobleaching (FRAP)

FRAP experiments used a Deltavision RT system (Applied Precision) with an Olympus IX70 microscope and a Cascade II EMCCD camera (Roper Scientific). Pre- and post-bleach images were acquired with 600 ms exposure time. Cell tips were bleached with a 488 nm laser pulse for 0.3 sec. Fluorescence recovery was measured either every 5 s for 5 min (Fig. 1 B, C, D, Fig. 3L), every 30 s for 30-50 min (Fig. 3I, Fig. S3 E, F) or every 60 s for 30 min (Fig. 3 J, K). Recovery curves were obtained using a script written in-house using Image Pro Analyzer (Media Cybernetics).

For immunostaining with anti-Tea1 antibody, wild-type and *tea1-GFP* cells were harvested by filtration and fixed in methanol at -80˚C for at least 10 min before being processed essentially as described [38]. Ten Z-sections at 0.6 μm spacing were captured with a Nikon TE300 inverted microscope connected to a CoolSnap HQ CCD camera (Photometrics). Z-sections were sum-projected onto a single plane and the total Tea1 signal at cell tips was calculated as the mean intensity within the region multiplied by its area, after subtracting the mean background intensity of a nearby area of comparable size.

For quantitative imaging of Tea1-GFP and GFP-Mod5 fluorescence, images were collected on a Nikon TE2000 inverted microscope connected to a Yokogawa CSU-10 spinning disk confocal head (Visitech) and an Andor Du888 EMCCD camera. Samples were illuminated with a 488 nm Coherent 20 mW laser. Cells were grown in EMM medium at 25ºC and imaged at room temperature in EMM 2% agarose pads [39]. Image acquisition, processing and analysis were carried out using

Metamorph software (Molecular Devices). Total cell fluorescence measurements were performed as described by Wu *et al.* [40], with some modifications. The entire volume of untagged cells and cells expressing Tea1-GFP and GFP-Mod5 was acquired in 12 Z-sections, 0.6 μm apart and sum-projected. Images were corrected for offset and uneven illumination. Cellular autofluorescence was corrected by subtracting the fluorescence of untagged cells from the fluorescence of Tea1-GFP and GFP-Mod5 expressing cells. Total fluorescence of Tea1-GFP and GFP-Mod5 expressing cells was converted to total number of molecules, using the results from quantitative immunoblotting. To measure fluorescence at the cell tip, the corrected images of Tea1-GFP and GFP-Mod5 described above were used. Because these images are projections, we distinguished membrane-associated tip signal from internal cytosolic signal within the cell-tip region, as follows (Fig. S3C): The area around the cell tip was divided into three rectangular regions of interest (ROIs) A, B, and C, each with dimensions r x $2r$, where r is the radius of the semi-circle that approximates the projection of the curved cell tip. ROI A contains only extracellular signal. ROI B contains extracellular signal, membrane-associated tip signal, and internal cytosolic signal, with the total contribution from the cell being a projection of a half-sphere with radius r. ROI C contains extracellular signal and internal cytosolic signal, with the total contribution from the cell being a projection of a cylinder with radius r and length r. Using geometry one can calculate the membrane-associated tip signal (i.e., the signal coming from an infinitely thin layer on the surface of the half-sphere described above) to be $Int_B - \frac{1}{3}Int_A - \frac{2}{3}Int_C$, where Int_{ROI} is the total integrated intensity of the named ROI (not shown). The number of Tea1-GFP and GFP-Mod5 molecules per cell tip was then calculated from the ratio of cell-tip fluorescence to total cell fluorescence, as described above. When GFP-Mod5 is 10x overexpressed, a significant amount of GFP-Mod5 is membrane-associated but not at cell tips, making the above method unsuitable for quantitation. Therefore to determine the cell tip-associated signal in these cells, instead of measuring ROI C *per se*, we measured the cytosolic signal in an internal region in the 3 middle z-sections and used this in subsequent calculations.

The number of Tea1-GFP molecules per microtubule-associated packet was determined using Z-sections of corrected images, using packets close to cell tips. To correct for background fluorescence we subtracted the fluorescence intensity of a region in the cytoplasm with the same area as the Tea1 packet. Number of molecules of Tea1 per packet was then determined from the ratio of packet fluorescence to total cell fluorescence as described above.

Delivery rate of Tea1 packets to the cell tip was determined by Tea1-GFP imaging using the spinning-disk confocal system described above. 8 Z-sections at 0.6 μm intervals were acquired every 5 sec for 10 min. The number of Tea1-GFP packets arriving per minute per cell tip was determined from kymographs.

For measurements of Tea1 residence time at the cell tip in *mod5Δ* mutants expressing GFP-Atb2 and Tea1-RFP (tdTomato; [12]), seven cells were analyzed from movies (Fig. S3G), after contact of a microtubule with the cell tip and Tea1

packet deposition. Tea1 residence time was determined as the interval between microtubule depolymerization and disappearance of Tea1 signal.

For measurements of cell tip-associated Tea1-GFP after treatment with actin- or microtubule-depolymerizing drugs, cells were mounted on EMM 2% agarose pads that also contained either 200 μM latrunculin B (LatB) or 262 μM methyl-benzimidazoyl-carbamate (MBC), respectively, with 1% DMSO final in the pads. DMSO alone was used as negative control. Preparations were imaged using the spinning-disk confocal system, and determination of cell tip-associated Tea1-GFP was as described above.

For high time-resolution Tea1-GFP imaging (i.e. Movie S1) the spinning-disk confocal system described above was used, streaming both time and z-sections. 8 Z-sections at 0.6 μm intervals were acquired every 1.65 sec for 5 min. In this instance the illumination intensity was intentionally kept at a high level to maximize signal, resulting in higher than normal photobleaching. The sequence was maximum-projected and compressed using Quicktime H.264 codec.

Biochemical methods

Total number of molecules per cell was determined as in Wu et al [40]. Recombinant Tea1-6xHis and 6xHis-MBP-Mod5(28-477aa) were used to quantify endogenous Tea1 and Mod5, respectively. Tea1-6xHis and 6xHis-MBP-Mod5(28-477aa) were expressed in *E. coli* BL21-CodonPlus (DE3)-RIL cells (Stratagene). Cells were grown in LB medium at 37˚C O/N, and diluted to OD_{600} 0.1. When cultures reached OD_{600} 0.5, protein expression was induced with 1mM IPTG and cells were grown O/N at 18˚C. Cells were harvested and 400 mg of pelleted cells were resuspended in 1600 μL of lysis buffer (6M guanidine-HCl, 25mM Tris-HCl pH8, 0.3M NaCl). Cells were disrupted by sonication, 4x 10 sec cycles at 30% output, duty cycle of 3, using a Branson sonicator. The soluble fraction was obtained after centrifugation at 4˚C at 55,000 rpm for 30 min in a TLS-55 rotor (Beckman). 2 mL of soluble extract were incubated with 200 μL slurry of nickel-activated His-bind Fractogel beads (Merck) for 60 min at 4˚ C. Beads were washed 4 times with 5 mL of wash buffer (8 M urea, 25 mM Tris-HCl pH 8, 0.3 M NaCl, 10mM imidazole). Proteins were eluted from beads with elution buffer (8 M urea, 25 mM Tris-HCl pH8, 0.3 M NaCl, 300 mM imidazole) and 2x Laemmli buffer (0.125M Tris-HCl pH8, 1% SDS, 10% glycerol) was added. Proteins were stored at -80˚C. The concentration of purified Tea1-6His and 6His-MBP-Mod5(28-477aa) was determined using a BSA calibration curve. To relate levels of recombinant protein to endogenous levels in fission yeast, *S. pombe* total cells extracts were prepared from 20 mL of exponentially growing culture at 25˚C in EMM2 supplemented with glutamate. Cells were harvested by centrifugation at 4000 rpm for 4 min at 20ºC. The cells were washed once with TBS and pelleted in a microfuge 1 min at 13,000rpm. The pellet was resuspended in a small volume of TBS and boiled for 5min. Cells were disrupted by bead-beating with 0.5 mm Zirconia beads in a Ribolyser at room temperature, using 2 cycles of 30 sec at 6.5. The supernatant was added to 2x Laemmli buffer, boiled for 5

min and centrifuged 10 min at 13, 000rpm, at room temperature. The supernatant was collected and protein concentration was determined by BCA assay. Cell extract corresponding to 0.5 to $2x10^6$ cells was loaded onto an 8% acrylamide gel. In the same gel were loaded 0.1 to 5ng of purified Tea1-6xHis or 6xHis-MBP-Mod5(28-477aa) mixed with 50µg of cells extract from *tea1*Δ and *mod5*Δ cells, respectively. Membranes were probed with polyclonal affinity purified anti-Tea1 or anti-Mod5 antibodies [11, 12]. Donkey anti-goat IRDye800 (Li-Cor Biosciences) was used as secondary antibody. Fluorescence intensity was quantified with the Odyssey scanner and software (Li-Cor Biosciences), and Tea1-6xHis and 6xHis-MBP-Mod5(28-477aa) calibration curves were used to determine endogenous levels of Tea1 and Mod5.

To measure *nmt* promoter-driven expression of Mod5 and GFP-Mod5 relative to endogenous Mod5 (e.g. under varying thiamine concentrations), quantitative immunoblotting of fission yeast extracts was performed using anti-Mod5 antibodies and Odyssey scanner and software as above.

For immunoprecipitation from native cell extracts, cells were frozen in liquid nitrogen and ground to a powder with a mortar and pestle, prior to resuspension in a buffer of 20 mM NaHEPES pH 7.5, 50 mM potassium acetate, 200 mM NaCl, 1 mM EDTA, 0.2% Triton X-100, and a protease inhibitor cocktail. After a clarifying centrifugation, the protein concentration was adjusted to the same concentration in all samples. 1 mL of each extract was incubated with 10 µL of Protein G-Dynabeads (Invitrogen) pre loaded with 10µg of anti-GFP, anti-FLAG (Sigma) or anti-tdimer2. Anti-FLAG2 antibodies were obtained from Sigma. Sheep affinity-purified anti-GFP, anti-tdimer2 and anti-Tea1 antibodies were generated in-house. After incubation with rotation for 2 hr at 4°C, beads were washed five times in 1 mL of buffer and resuspended in Laemmli sample buffer for SDS-PAGE and western blotting.

Model formulation and computational implementation

Formation of the cell tip Tea1-Mod5 cluster-networks in fission yeast is dependent on the dynamics of microtubule bundles (further, for simplicity, microtubules; MTs) that in wild-type cells deliver Tea1 on their growing ends to the plasma membrane [6, 10]. The act of Tea1 transmission from the MT ends to the membrane is thought to occur during MT catastrophe which may be induced by the mechanical interaction of MTs with the cell-wall-supported membrane [23, 41]. In the absence of Mod5, a low-level amount of Tea1 is detectable at cell tips as a residue of the MT-delivered packets; however, formation of proper cluster-networks characteristic of wild-type cells requires Mod5. Time-lapse analysis of Tea1 behavior in *mod5*Δ cells suggests that membrane residence time is not a kinetic bottleneck for Tea1 accumulation. Indeed, individual Tea1 packets arrive approximately every minute (**Fig. S3D**; additional data not shown) and the material of the packet remains detectable on the membrane for ~ 2 min (**Fig. S3G**). Given the finite size and further diffusive spread of deposited packets, successively deposited Tea1 molecules ought to interact during their lifetime on the membrane. Absence of Tea1 accumulation in this

case suggests that the characteristic time of Tea1 spontaneous polymerization is > 1 min. We thus hypothesize that Mod5 directly or indirectly accelerates Tea1 polymerization via a reversible mechanism presented below.

On the basis of observations in *mod5*Δ cells we assume that, even in the absence of Mod5, Tea1 has an appreciable affinity for the membrane, where it resides as a membrane-associated species, T_{mem}. T_{mem} recycles to the cytoplasm with rate k_2 and can reversibly interact (k_1, k_{-1}) with membrane-associated Mod5, M_{mem}. When interacting with Mod5 in a bimolecular complex Tea1·Mod5, *TM*, Tea1 can reversibly form bonds (k_3, k_{-3}) with Tea1 molecules incorporated into other *TM* complexes or the polymeric networks, T_{pol}. As specified earlier, this interaction need not be direct and may involve other molecules that bridge individual Tea1 monomers. To enable Tea1 polymerization beyond dimers and simple linear chains, we assumed that each Tea1 monomer can form at least three Tea1-Tea1 bonds and any Tea1 molecule forming at least one such bond is considered a part of a polymeric network, independently of the global connectivity. Local connectivity $n = 3$ provides compact hexagonal packaging in 2D and is commonly suggested for biological polymeric lattices on membranes by direct experimental observation [42, 43] and inference on the basis of valences of known molecular interactions [44, 45].

All Mod5 molecules bound to the polymeric Tea1 are assumed to be within the polymer, M_{pol}. For example, to describe the initiation of polymerization, we explicitly consider dimerization of *TM* (see Fig. 2C, ii) as follows: $TM + TM \leftrightarrow 2T_{pol} + 2M_{pol}$.

According to our hypothesis, Mod5 readily associates with and dissociates from the polymeric network, resulting in diffusion-capture dynamics of individual Mod5 molecules. We posit that Tea1 monomer can form a bond with only one molecule of Mod5, and thus at any location within the polymer, $T_{pol} \geq M_{pol}$. Since lifting this restriction resulted in model behavior that contradicted experimental results (data not shown), we adopted the above inequality and the underlying stoichiometric assumption as the most consistent with the available data.

In considering dissociation of Tea1-Tea1 bonds, we recognized three distinct potential outcomes: 1) dissociation from the polymer of a Tea1 monomer, T_{mem}, 2) dissociation of a *TM* complex from the polymer, or 3) change in the Tea1 network connectivity. We found in our simulations that spontaneous (not mediated by Mod5) "shedding" of Tea1 from the polymer (outcome 1), i.e., $T_{pol} \rightarrow T_{mem}$, does not alter the model behavior qualitatively. Since we do not have any experimental evidence supporting this reaction, we omitted it from the reaction mechanism to reduce the model complexity. Instead, reversible dissociation of *TM* (outcome 2) is taken to be the main pathway for polymer remodeling and fluidity, and the probability of this dissociation is proportional to the local concentration of polymerized Mod5, M_{pol}. On the other hand, according to our definitions, breaking of Tea1-Tea1 bonds without dissociation of *TM* (outcome 3, see Fig. 2E) does not change T_{pol} and M_{pol} concentrations. Thus formation and dissociation of Tea1-Tea1 bonds that alter only the connectivity of the network are events not registered within our framework. The

advantage of this approach, similar to that commonly followed in modeling actin dynamics (see, e.g., [46] and references therein), is that it avoids the necessity to consider an infinite number of molecular species representing all possible stoichiometric complexes [47]. The introduced coarse-graining, however, also averages out all fluctuations in the spatial density of Tea1 within the cluster-networks. Concentration profiles obtained in our simulations thus could be considered as the average of many stochastic realizations of the possible microscopic configurations, such as that shown in Fig. 2E, and should be compared with population-averaged experimental data as is shown in Fig. 3E.

Using the mass-action rate law formalism, the above reaction mechanism can be summarized as a system of five coupled reaction diffusion equations:

$$\begin{aligned}
\dot{M}_m &= k_{-1}TM - k_1 T_m \cdot M_m + k_{-1}M_p - k_1 M_m \cdot (T_p - M_p) + D_m \Delta M_m \\
\dot{T}_m &= F(r, T_{cyt}) + k_{-1}TM - k_1 T_m \cdot M_m - k_2 T_m + D_m \Delta T_m \\
\dot{TM} &= k_1 T_m M_m - k_{-1}TM - 2k_3 TM^2 + k_{-3}M_p - k_3 TM \cdot T_p + D_m \Delta TM \\
\dot{M}_p &= 2k_3 TM^2 + k_3 TM \cdot T_p - k_{-3}M_p + k_1 M_m \cdot (T_p - M_p) - k_{-1}M_p + D_p \Delta M_p \\
\dot{T}_p &= 2k_3 TM^2 + k_3 TM \cdot T_p - k_{-3}M_p + D_p \Delta T_p
\end{aligned} \qquad (1)$$

where subscripts *mem* and *pol* have been reduced respectively to *m* and *p* for brevity. To enforce the inequality $M_{pol} \le T_{pol}$, the incorporation of monomeric Mod5 into the polymeric network is given by $k_1 M_m \cdot (T_p - M_p)$. MT-based delivery of Tea1 to the membrane is represented by the term $F(r, T_{cyt})$, which is a function of the spatial location on the membrane and the amount of Tea1 in the cytoplasm (see below). As we do not explicitly consider cytoplasmic Tea1 and its interaction with MTs, all species in our model are taken to be membrane-bound, and their concentrations are computed per volume V_m, which, following the approach developed in [31, 48], represents a thin ($h \approx 10$ *nm*) layer of the cytoplasm directly above the membrane. The diffusion coefficient of monomeric Mod5 was estimated from the *tea1*Δ FRAP data to be $D_m \approx 0.05 \mu m^2 / s$, and diffusion coefficients of T_m and *TM* are taken to be equal to D_m for simplicity. Tea1-Mod5 polymer is assumed to diffuse much slower, and its diffusivity was varied in the range $D_p = 10^{-3} - 10^{-6}\ \mu m^2 / s$ to achieve the best fit with the experimental data.

From our measurements we estimated an average cell in the growing population to be represented by a spherocylinder with the diameter $4\,\mu m$ and length $11\,\mu m$, resulting in a geometric cell volume $V = 121.5 \mu m^3$ and cell surface $S = 138 \mu m^2$ (thus $V_m = S \cdot h = 1.38 \mu m^3$ so that 1000 molecules per V_m result in a concentration of $1.2 \mu M$). In our simulations we considered *de novo* formation of the cluster-networks; therefore initially all Tea1 (8000 molecules/cell) was assumed to be in the cytoplasm. In experiments we found that a certain proportion of cellular Mod5 does not reside on the plasma membrane (fluorescent signal was detected in the cytoplasm and vacuoles). According to our FRAP data, however, these additional

pools do not dynamically exchange with the membrane and thus for the purposes of modeling can be disregarded. The unknown initial concentration of membrane-bound Mod5, M_{mem}, was varied in our simulations to fit the data.

Our experimental observations suggest that the number of Tea1 molecules per packet delivered by MT ends is inversely proportional to the amount accumulated at the cell tips and thus should be directly proportional to the cytoplasmic pool of Tea1. Therefore we choose a Tea1 input function in the form:

$$F(r,T_{cyt}) = k_0 \left(1 - \int_S T_{mem} dS / T_{tot}\right) f(r), \qquad (2)$$

where T_{tot} is the total cellular Tea1, the integral represents the overall amount of Tea1 on the membrane, k_0 is the maximal intensity of Tea1 input at the initiation of cluster-network formation and $f(r)$ is the nondimensional spatial function with values in the interval (0,1) that represents the MT target zone. Our measurements in the wild-type cells demonstrated that at steady state (ss), Tea1 is delivered to a tip with the intensity 80 molecules/min (or $k_{ss} \sim 0.01 \mu M / s$ for the chosen cell geometry). Therefore according to (2), the expected maximal intensity of Tea1 input is $k_0 = k_{ss} / T_{cyt}^{ss}$, where T_{cyt}^{ss} is the fraction of Tea1 in the cytoplasm at the steady state.

As the spatial probability density function of Tea1 delivery is difficult to measure experimentally, we assumed that MT ends visit the surface of the hemispherical cell tips with uniformly distributed probability everywhere except the sphere-to-cylinder transition zone, where the probability of delivery rapidly falls, resulting in the profile shown in Fig. 4C. In our simulations, Tea1 input was achieved either by a continuous source term (2) or by discrete packets deposited at uniformly distributed random locations with a 1 min interval. The size of the packet, chosen after fitting the model to be 116 molecules at the beginning of the simulation, was progressively decreased proportionally to the Tea1 accumulation at the tips as specified by (2). Simulations with discrete packet delivery result in a stochastic steady state that changes perpetually as MT-end packets continue to arrive at the tips. Averaging of these states over a time period or over several independent realizations (equivalent to a population average) results in a recovery of the time-independent steady state obtained in the simulations with the continuous input function (2). This proves the equivalence of the two methods; however, continuous input has an advantage of providing a population-averaged steady state and thus saves computational costs associated with averaging over multiple stochastic realizations.

Model (1), with the Tea1 input function (2) and parameters defined as described above and summarized in the Table S2, was numerically integrated using a standard finite difference algorithm implemented as a custom C code. To leverage cylindrical symmetry of the system with continuous Tea1 input, in addition to 2D simulations we also performed simulations on a 1D domain representing circumference of the plasma membrane cross-section. Simulations in 1D, which were considerably less computationally expensive, showed results in a good agreement

with the 2D simulations. In most cases (except FRAP simulations, see below) it was possible to further reduce computational costs by considering only one half of a cell.

FRAP Modeling

In our FRAP experiments, photobleaching was achieved using a laser with Gaussian intensity profile characterized by the beam diameter at the half maximal intensity ($1.42\mu m$ for the full-tip photobleaching experiments and $0.774\mu m$ for the half-tip measurements). Beam intensity profile was converted into the efficiency of photobleaching using the fluorescence intensity immediately after the bleaching flash. To model the recovery of fluorescence, we introduced additional variables representing bleached species with chemical properties identical to those of their unbleached counterparts. For example, for the concentration of the bleached Tea1 molecules incorporated into the polymeric network, T_{pol}^B, we get:

$$\dot{T}_p^B = k_3 TM^B(2TM^B + TM) + k_3 TM^B(T_p + T_p^B) - k_{-3} M_p \frac{T_p^B}{T_p + T_p^B} + D_p \Delta T_p^B$$

Initial concentrations for the FRAP modeling were computed by applying a bleaching efficiency profile to the steady state solution of (1). Two separate input functions for the bleached and unbleached Tea1 were introduced to take into the consideration their MT-mediated membrane-cytoplasmic shuttling. FRAP simulations were performed using the whole-cell model to account for the fluorescence recovery due to the exchange with the unbleached cluster-network at the opposite cell tip.

Parametric robustness, model validation and evaluation of confidence intervals

Extensive variation of model parameters demonstrated that in the presence of continuous MT-associated flux of incoming cytoplasmic Tea1 and non-zero initial concentrations of Tea1 and Mod5, our model always converges to a spatially heterogeneous steady state characterized by a Tea1 accumulation profile with a width approximately defined by that of the input function $f(r)$ and an amplitude that varies widely with the model parameters. Thus the emergence of a structure is guaranteed by the presence of the MT-associated flux of Tea1. Since the required threshold for a functional Tea1 polarity landmark is not known, it was not possible to directly compute the parameter domain corresponding to a functional landmark. However, our results in cells with substantially reduced Mod5 expression suggest that even a fraction of wild-type Tea1 accumulation is sufficient to sustain polarized vegetative growth.

In order to estimate the predictive power of our model, we constructed strains with altered expression level of Mod5, measured the resulting levels and used these data to calculate Tea1:Mod5 tip ratios (with and without including concentrations of free proteins) and FRAP curves for full-tip photobleaching of both Tea1-GFP and GFP-Mod5. The good-quality fit with experimental data seen on Figs. 3I-L was achieved by fitting only the efficiency of photobleaching (see above), the only

parameter of the *in silico* FRAP simulation that can be neither independently experimentally measured nor predicted from our model. Not fitting this parameter would have resulted in experimental and theoretical FRAP curves starting at $t = 0$ with arbitrarily offset percent recovery, thus obscuring otherwise good correspondence between the theory and experiment.

To evaluate the degree of uncertainty associated with our model predictions for cells with altered levels of Mod5, an heuristic approach was taken. To compute envelopes shown as shaded areas in Figs. 3I-L, in each case, FRAP curves were calculated for 16 perturbed parameter sets in which one of the six reaction rates or two protein concentrations were multiplied or divided by a fixed factor. From our experimental results, we estimated that levels of protein expression are measured with ~20% variation, thus a factor of 1.2 was chosen to reflect uncertainty based on protein level determination. Since no such estimates were available for reaction rates, a heuristic factor of 2 was chosen. The resulting envelope was computed as an area between the minimal and maximal values of percent recovery at each time point. Position of the prediction corresponding to the optimized parameter set (shown by solid line in figures) in relation to this envelope and its width indicate that the model is robust to parameter variation. As essentially all perturbed parameter sets appeared to predict experimental results worse than the optimized set, these calculations also supported our confidence in the identified optimized parameter set as the set genuinely representing the system dynamics.

A similar approach was taken to compute confidence intervals for comparison of the predicted and experimentally-measured profiles of steady state Tea1 tip accumulation shown in Fig. 3G. To calculate the experimental profile (dashed line), a semicircular path was drawn through the averaged Tea1-GFP tip signal in Fig. 3E, so that the center of the path went through the point of maximal intensity, and the intensities along this path were collected as an approximation of the average intensity of Tea1-GFP signal on the surface of the membrane. As the image presented in Fig. 3E was obtained by averaging 60 randomly selected cell tips, the size of the sample was found insufficient to fully cancel noise inherent in individual cell images. To estimate the residual level, the following statistical procedure was implemented. A pool of 1000 independent stochastic realizations of steady-state Tea1 accumulation was computed using stochastic packet-based delivery. 60 independent realizations were randomly selected from the pool 100 times and used to produce 100 averaged Tea1 profiles. To define the envelope shown as the shaded area in Fig. 3G, 5% and 95% percentiles at each point along the cell tip were selected from these 100 averaged profiles.

Calculation of incorporation efficiency and polymerization reaction flux

To calculate the proportion of the MT-deposited Tea1 that becomes incorporated into the polymeric network, we utilized a strategy very similar to that of FRAP simulation. While at steady state in a simulation with Tea1-packet input, all Tea1-containing species were “bleached” *in silico* simultaneously everywhere on the

membrane. In order to calculate incorporation efficiency for a specific sub-area of the total MT target zone, only the packets delivered within this sub-area were considered as contributing "unbleached" Tea1. Obviously, given the artificial nature of "bleaching" in this computational experiment, all Tea1 recycled back to the cytoplasm automatically becomes "unbleached". Monitoring the kinetics with which the "unbleached" material accumulates within the cluster-networks allows us to compute the average incorporation efficiency within the chosen sub-area. Indeed, averaged over multiple stochastic realizations, accumulation curves could be fitted to single-exponential functions of the type $A(1-\exp(-t/\tau))$. The absolute rate of incorporation averaged per tested area (in molecules per min) is then given simply by A/τ. Normalizing this value by the computed total rate of Tea1 delivery at steady state and further by the ratio of the tested area to the total area of the MT target zone gives the desired incorporation efficiency, as a fraction of 1. Using this approach and the entire MT target zone as the area of interest, we calculated an average incorporation efficiency of 0.33 for the model parameters presented in the Table S2. To test the hypothesis that the incorporation efficiency depends on the local concentration of already-deposited Tea1, and thus on the location within the MT target zone, we subdivided the entire MT target zone into two sub-zones with equal areas, the disc-shaped center and the periphery, with annular geometry. Our analysis demonstrated that the efficiency of incorporation in the central zone (0.44) is exactly twice that (0.22) at the periphery (see Fig. S4A, iii). As expected, the mean of the two recovers the computed earlier average efficiency of 0.33. To further characterize this spatial dependence, we subdivided the entire MT target zone as shown in Fig. S4A (i) and (ii) and computed the respective efficiencies. The results of these calculations are summarized in the graph in Fig. S4A, where the underlying continuous probability density function is reconstructed approximately and shown schematically as a dashed line. This shows that the incorporation efficiency is nearly optimal (~80%) at the very center of the cluster-network and drops by approximately four-fold at the rim of the MT target zone.

Spatial distribution of the T_{pol} polymerization reaction flux within the cluster-network shown in Fig. 4D was computed as follows. From (1), steady state reaction flux for T_{pol} was computed as $2k_3TM^2 + k_3TM \cdot T_p - k_{-3}M_p$, where all variables are respective local steady-state concentrations. In the presence of spatial gradients associated with the cluster-network concentration profile, the steady-state diffusive flux of T_{pol} is non- zero ($D_{pol}\Delta T_{pol} \neq 0$). At the same time, at steady state, $dT_{pol}/dt = 0$ and thus, as follows from equations (1), diffusive flux must be exactly compensated by the local reaction flux.

SUPPLEMENTAL REFERENCES

33. Tran, P.T., Marsh, L., Doye, V., Inoue, S., and Chang, F. (2001). A mechanism for nuclear positioning in fission yeast based on microtubule pushing. J Cell Biol *153*, 397-411.
34. Busso, D., Delagoutte-Busso, B., and Moras, D. (2005). Construction of a set Gateway-based destination vectors for high-throughput cloning and expression screening in Escherichia coli. Anal Biochem *343*, 313-321.
35. Matsuyama, A., Shirai, A., Yashiroda, Y., Kamata, A., Horinouchi, S., and Yoshida, M. (2004). pDUAL, a multipurpose, multicopy vector capable of chromosomal integration in fission yeast. Yeast *21*, 1289-1305.
36. Moreno, S., Klar, A., and Nurse, P. (1991). Molecular genetic analysis of fission yeast Schizosaccharomyces pombe. Methods Enzymol *194*, 795-823.
37. Sambrook, J., and Russell, D.W. (2001). Molecular Cloning: A Laboratory Manual, 3rd Edition, (New York: Cold Spring Harbor Laboratory Press).
38. Sawin, K.E., and Nurse, P. (1998). Regulation of cell polarity by microtubules in fission yeast. J Cell Biol *142*, 457-471.
39. Sawin, K.E. (1999). GFP fusion proteins as probes for cytology in fission yeast. Methods Cell Biol *58*, 123-138.
40. Wu, J.Q., McCormick, C.D., and Pollard, T.D. (2008). Chapter 9: Counting proteins in living cells by quantitative fluorescence microscopy with internal standards. Methods Cell Biol *89*, 253-273.
41. Tischer, C., Brunner, D., and Dogterom, M. (2009). Force- and kinesin-8-dependent effects in the spatial regulation of fission yeast microtubule dynamics. Mol Syst Biol *5*, 250.
42. Brodsky, F.M., Chen, C.Y., Knuehl, C., Towler, M.C., and Wakeham, D.E. (2001). Biological basket weaving: formation and function of clathrin-coated vesicles. Annu Rev Cell Dev Biol *17*, 517-568.
43. Scott, F.L., Stec, B., Pop, C., Dobaczewska, M.K., Lee, J.J., Monosov, E., Robinson, H., Salvesen, G.S., Schwarzenbacher, R., and Riedl, S.J. (2009). The Fas-FADD death domain complex structure unravels signalling by receptor clustering. Nature *457*, 1019-1022.
44. Shimizu, T.S., Le Novere, N., Levin, M.D., Beavil, A.J., Sutton, B.J., and Bray, D. (2000). Molecular model of a lattice of signalling proteins involved in bacterial chemotaxis. Nat Cell Biol *2*, 792-796.
45. Sola, M., Bavro, V.N., Timmins, J., Franz, T., Ricard-Blum, S., Schoehn, G., Ruigrok, R.W., Paarmann, I., Saiyed, T., O'Sullivan, G.A., et al. (2004). Structural basis of dynamic glycine receptor clustering by gephyrin. EMBO J *23*, 2510-2519.
46. Carlsson, A.E., and Sept, D. (2008). Mathematical modeling of cell migration. Methods Cell Biol *84*, 911-937.
47. Goldstein, B., and Perelson, A.S. (1984). Equilibrium theory for the clustering of bivalent cell surface receptors by trivalent ligands. Application to histamine release from basophils. Biophys J *45*, 1109-1123.
48. Kholodenko, B.N., Hoek, J.B., and Westerhoff, H.V. (2000). Why cytoplasmic signalling proteins should be recruited to cell membranes. Trends Cell Biol *10*, 173-178.
