# Supplementary figures and images for: A Catalytic Role for Mod5 in the Formation of the Tea1 Cell Polarity Landmark
Source: Curr Biol. 2010 Oct 12;20(19):1752–7. doi: 10.1016/j.cub.2010.08.035 (PMC3094757; doi:10.1016/j.cub.2010.08.035)

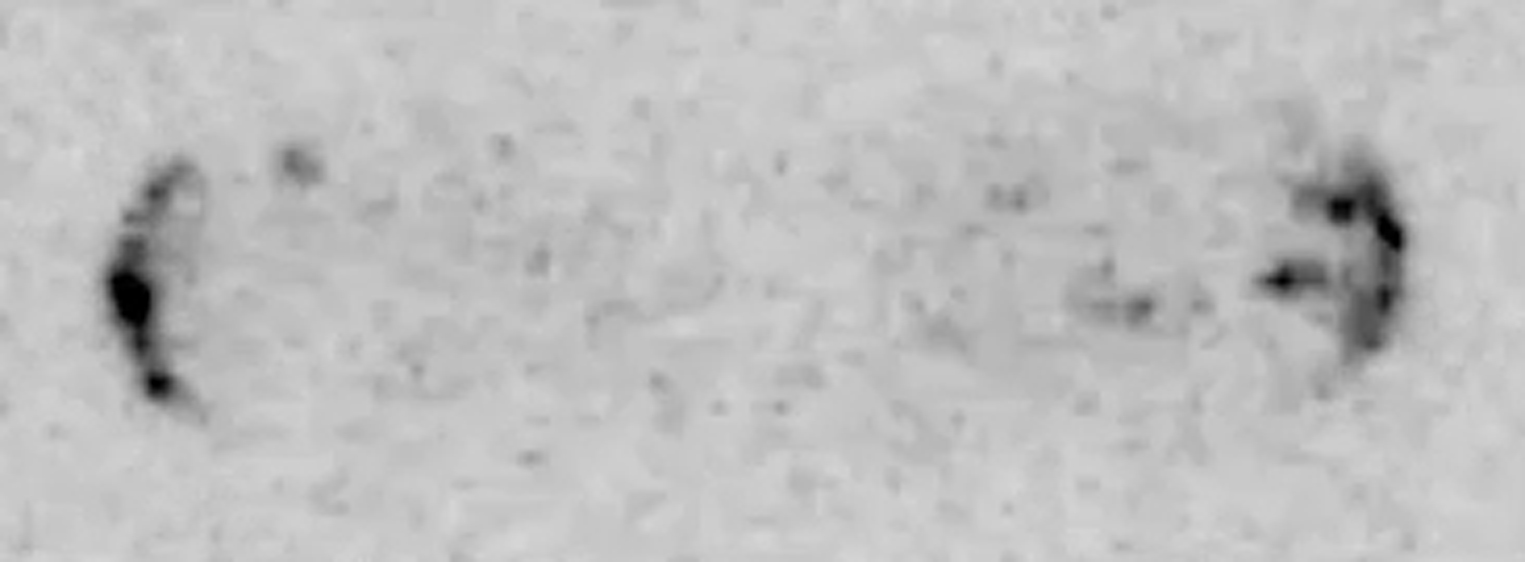

Supplement: Movie S1. High Time-Resolution Imaging of Tea1-GFP — Spinning-disk confocal imaging of Tea1-GFP in packets and at cell tips in a wild-type cell over a 5 min period (time compression ∼50×). Maximum projection of eight Z sections is shown. To increase signal, we intentionally kept illumination intensity high relative to conventional imaging; as a result some photobleaching is observed. Note that Tea1 packets appear to lose individual identity over time. We interpret variations in intensity at cell tips as the superposition of incoming Tea1 packets and dynamic rearrangements of Tea1 in cluster networks (see main text). [file mmc2.jpg]
